# Efecto de la inmunoterapia con alérgenos específicos en pacientes pediátricos con asma atendidos en una institución de salud de Colombia

**DOI:** 10.7705/biomedica.5673

**Published:** 2021-09-22

**Authors:** Jorge Andrés Puerto, Susana Uribe, Víctor Calvo, Ricardo Cardona

**Affiliations:** 1 Grupo de Alergología Clínica y Experimental, Facultad de Medicina, Universidad de Antioquia, Medellín, Colombia Universidad de Antioquia Grupo de Alergología Clínica y Experimental Facultad de Medicina Universidad de Antioquia Medellín Colombia

**Keywords:** inmunoterapia, asma, pediatría, ácaros, espirometría, rinitis, Immunotherapy, asthma, pediatrics, mites, spirometry, rhinitis

## Abstract

**Introducción.:**

El asma es una enfermedad crónica y potencialmente grave. El 80 % de los casos es de origen alérgico, por lo cual la inmunoterapia específica con alérgenos es una alternativa terapéutica que modula el curso natural de la enfermedad.

**Objetivo.:**

Evaluar el impacto de la inmunoterapia en pacientes pediátricos con asma atendidos en una institución de salud de Colombia.

**Materiales y métodos.:**

Se hizo un estudio observacional descriptivo con componente analítico de corte transversal. Se incluyeron 62 pacientes con diagnóstico de asma alérgica sensibilizados a ácaros del polvo y en tratamiento, mínimo, con seis dosis de inmunoterapia contra ácaros. El efecto del tratamiento se evaluó mediante la escala de puntuación del ACT *(Asthma Control Test),* la escala de tratamiento de la GINA *(Global Initiative for Asthma)* y la espirometría.

**Resultados.:**

La puntuación de la prueba ACT antes del inicio de la inmunoterapia, correspondía a 30 % de pacientes con asma no controlada, 28 % con buen control y 4 % con asma totalmente controlada. Entre los pacientes con asma no controlada, el 46,7 % logró un buen control y el 23,3 % alcanzó un control total. En cuanto a la percepción de los pacientes sobre la mejoría con la inmunoterapia, el 9,75 % percibió una mejoría menor del 50 %, el 45,2 %, una entre el 50 y el 90 %, en tanto que el 41,9 % refirió una igual o mayor del 90 %. No se encontraron cambios significativos en los valores del volumen espiratorio forzado en un segundo (VEF1) en las espirometrías.

**Conclusiones.:**

Se observaron cambios significativos en los puntajes del ACT y en la percepción de mejoría de la enfermedad en la población tratada con inmunoterapia específica para ácaros, es decir, que esta tendría un efecto beneficioso en el curso natural de la enfermedad

## Abstract (en)

**Introduction:**

Asthma is a chronic and potentially serious disease and 80% of the cases have an allergic etiology. In this sense, allergen-specific immunotherapy is an alternative that modulates the natural course of the disease.

**Objective:**

To evaluate the impact of immunotherapy in pediatric asthma patients treated at a health institution in Colombia.

**Materials and methods:**

We conducted an observational descriptive study with an analytical cross-sectional component. Sixty-two patients diagnosed with allergic asthma sensitized to dust mites and treated with at least 6 doses of mite immunotherapy were included. We assessed the impact of immunotherapy using the Asthma Control Test (ACT), the Global Initiative for Asthma (GINA) treatment scale, and spirometry values.

**Results:**

The ACT score before the start reported 30% of patients with uncontrolled asthma, 28% with good control, and 4% with totally controlled asthma. Of the patients with uncontrolled asthma, 46.7% achieved good control and 23.3% total control. Regarding patients' perception of improvement with the immunotherapy, 9.75% perceived a response of less than 50%, 45.2% one between 50% -90%, and 41.9% reported response equal to or greater than 90%. No significant changes in FEV1 values were found in spirometry.

**Conclusions:**

Significant changes in the ACT scores and the perception of disease improvement were observed in the population evaluated with specific mite immunotherapy, i.e., it had a positive impact on the natural course of the disease.

El asma es un problema de salud mundial, una enfermedad crónica y potencialmente grave que afecta, aproximadamente, a 300 millones de personas de todas las edades, países y grupos étnicos ^1^. En Colombia, la prevalencia aumentó del 10 % en el 2004 ^2^ al 12,1 % en el 2010 ^3^.

Sus síntomas incluyen disnea, tos, opresión en el pecho y sibilancias asociadas con disminución del flujo de aire espiratorio con reversibilidad en la espirometría previa al uso de broncodilatadores y la posterior ^4^. Hay diferentes factores asociados con el desarrollo de la enfermedad y su variabilidad en el tiempo y según la población, entre ellos, los factores genéticos que, sin embargo, por sí solos no son determinantes ^5^.

La atopia se reconoce como el principal factor de riesgo no modificable para el desarrollo del asma. Se estima que entre el 60 y el 80 % de los pacientes asmáticos puede tener una sensibilización mediada por la inmunoglobulina E (IgE) a alguna sustancia del medio ambiente, lo cual sugiere una fuerte relación entre dicha sensibilización y el desarrollo de la enfermedad ^6^.

Esta reacción inflamatoria mediada por IgE de tipo 2 es crucial en el asma alérgica ^7^, por lo que la inmunoterapia con extracto alergénico es la única que modifica el curso natural de la enfermedad, cambiando la reacción inflamatoria a una mediada por Th1 ^8^, con lo que se logra una mejoría clínica duradera hasta en el 70 % de los pacientes tratados, porcentaje que varía según el órgano afectado y el extracto de alérgeno utilizado ^9^.

En Colombia, ya se hizo un estudio para evaluar el impacto de la inmunoterapia en pacientes con asma alérgica, sin embargo, al año de seguimiento hubo limitaciones debido al reducido tamaño de la muestra ^10^. En este contexto, en este estudio se evaluó el impacto de la inmunoterapia subcutánea en una muestra de pacientes pediátricos con asma atendidos en un servicio hospitalario de referencia en alergología.

## Materiales y métodos

Se hizo un estudio observacional descriptivo con componente analítico de corte transversal. Se reclutaron 62 pacientes del proyecto *Research About Tropical Trends in Asthma* (RATTA) ^11^ llevado a cabo en el 2016, el cual incluyó 150 pacientes de Medellín con edades entre los 6 y los 17 años y diagnóstico clínico de asma basado en la escala de la *Global Initiative for Asthma,* (GINA) ^4^ y confirmado mediante espirometría antes y después de usar el broncodilatador.

El estudio buscaba hacer una caracterización clínica y sociodemográfica de los pacientes con asma y de los factores asociados con la enfermedad, como la cuantificación de IgE y la sensibilización a alérgenos. Dicho estudio se continuó para determinar cuáles de estos pacientes recibían inmunoterapia y cuál era su impacto en la enfermedad, por lo que la selección de los pacientes obedeció a la conveniencia clínica ^12^.

Se hizo un análisis retrospectivo de las historias clínicas de los participantes en el proyecto RATTA; los criterios de inclusión fueron el haber recibido, por lo menos, seis dosis de inmunoterapia con extracto de dos ácaros *(Dermatophagoides farinae* y *Dermatophagoides pteronyssinus)* de polvo doméstico *(House Dust Mite,* HDM) o de tres *(D. farinae, D. pteronyssinus* y *Blomia tropicalis),* administradas por vía subcutánea.

Se usaron extractos alergénicos polimerizados inyectables (Alxoid®) o extractos alergénicos glicerinados por vía sublingual (Oraltek®), ambos de Inmunotek. Para la evaluación de las historias clínicas, se usó el *Asthma Control Test*TM (ACT) en la versión pediátrica para los pacientes de 4 a 11 años de edad.

Este consiste en cinco preguntas relacionadas con la frecuencia de los síntomas asmáticos (sibilancias, tos nocturna y diurna, tos con la actividad física, despertares nocturnos) y el uso de medicación de rescate requerida por el paciente en las cuatro semanas previas; un puntaje de 25 corresponde al asma totalmente controlada, uno entre 20 y 16, a la bien controlada, y uno de 5 a 15, a la no controlada ^13^.

La puntuación en la escala de tratamiento de la GINA evalúa la medicación necesaria para el control del asma y clasifica a los pacientes en cinco grados con un puntaje de 1 a 5. Los grados 1 y 2 corresponden a pacientes con medicación para controlar el asma leve y, el grado 5, a aquellos que necesitan medicamento para el asma grave. Los datos obtenidos de las historias antes de empezar la inmunoterapia se superpusieron a los de las escalas y así se asignó el puntaje en el ACT y el grado de tratamiento de la GINA, según lo explicado. Los pacientes seleccionados debían tener datos de espirometría previos a la inmunoterapia.

**Figura 1:**
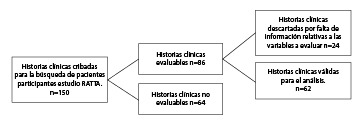
Estudio RATTA *(Research About Tropical Trends in Asthma)*

El estudio RATTA *(Research about Tropical Trends in Asthma)* contó con 150 pacientes. Se hizo una revisión de las historias clínicas que cumplían con los criterios establecidos; se encontraron 86 historias evaluables, de las cuales 24 no tenían datos de espirometría ni número mínimo de dosis deinmunoterapia. Es decir, 62 cumplían con los criterios mencionados y eran evaluables (figura 1), por lo que se les invitó a formar parte del estudio mediante llamadas telefónicas y se evaluó su estado de salud en el momento del estudio. Se excluyeron los pacientes con otras enfermedades pulmonares y quienes no cumplían con los requisitos mencionados. Se excluyeron los pacientes con otras enfermedades pulmonares y quienes no cumplían con los requisitos mencionados. A todos los participantes se les explicaron los riesgos y beneficios, se les pidió el consentimiento informado y sus respuestas al cuestionario modificado y abreviado del Departamento Administrativo Nacional de Estadísticas (DANE) para obtener información sobre las condiciones de la vivienda y la interacción con factores como el tabaquismo. Se midió la talla, el peso y se calculó el índice de masa corporal (IMC).

La evaluación y clasificación de la rinitis se hizo según la puntuación de la *Allergic Rhinitis and its Impact on Asthma* (ARIA) ^14^. La reacción a la inmunoterapia se evaluó con base en los puntajes del ACT y la escala de tratamiento de la GINA y los resultados de la espirometría previos a su inicio y en el momento de la evaluación clínica del estudio para determinar el número de dosis que llevaban hasta ese momento. Se les preguntó por su percepción de la reacción a la inmunoterapia mediante una escala visual análoga y se clasificaron como, sin mejoría, cuando el puntaje era menor del 50 %, como mejoría parcial, cuando era del 50 al 90 % y, como mejoría total, cuando era de más del 90 %.

Se evaluó el efecto de la inmunoterapia específica con alérgenos, teniendo en cuenta la variable del tiempo para un mínimo de seis dosis y un máximo de 54 dosis. El efecto positivo de la inmunoterapia se definió como un aumento en el puntaje del ACT y una disminución en el grado de la GINA entre el momento de evaluación del paciente y lo registrado en la historia clínica antes de iniciar la inmunoterapia. El efecto negativo se determinó cuando no se registró ningún cambio en el puntaje del ACT o el grado de la GINA o cuando hubo una disminución en el puntaje del ACT y un aumento en el grado de la GINA.

Por último, la espirometría previa al broncodilatador y la posterior estuvieron a cargo de personal entrenado utilizando un espirómetro Easy on-PC™ previamente calibrado y con un equipo capaz de definir los valores de límite inferior de normalidad y los parámetros del estudio PLATINO (Suramérica). Todos los datos se consignaron en la historia clínica del paciente. La recolección de datos, el análisis de las historias clínicas que cumplían con los criterios de inclusión y las pruebas, se realizaron entre mayo del 2018 y enero del 2020.

Este estudio fue aprobado por el comité de ética de la IPS Universitaria, sede Medellín, en el 2018, y se ajusta a las normas éticas estipuladas en la Declaración de Helsinki, además de las de la Organización Mundial de la Salud (OMS) y la Resolución 8430 de 1993 del Ministerio de Protección Social. Dado que los procedimientos hacen parte de la evaluación clínica rutinaria, el estudio no representó un riesgo adicional al que tiene comúnmente un paciente que asiste a la consulta de alergología clínica.

### Plan de análisis estadístico

Para el análisis descriptivo de los aspectos sociodemográficos y clínicos de los pacientes, se utilizaron distribuciones absolutas y relativas, e indicadores de resumen como la media aritmética, la desviación estándar, y valores mínimos y máximos. Se estableció el criterio de normalidad de algunas variables mediante la prueba de Shapiro-Francia.

Para evaluar el impacto de la inmunoterapia por medio de la puntuación del ACT, de los grados de la GINA y de la espirometría previa y la posterior al broncodilatador, se utilizó la prueba de los rangos con signo de Wilcoxon complementada con el tamaño de efecto del coeficiente de correlación biserial, antes y después de la inmunoterapia.

Para evaluar la decisión clínica basada en el ACT, se utilizó la prueba de McNemar-Bowker; se consideró estadísticamente significativo un valor de p<0,05.

En cuanto a la relación de las condiciones de la inmunoterapia según la mejoría del paciente después de aplicarla, se utilizó la prueba de ji al cuadrado de independencia de la razón de verosimilitud, y la fuerza de asociación se evaluó con base en la razón de proporción (RP), con sus respectivos intervalos de confianza del 95 % (IC95%).

## Resultados

### Aspectos sociodemográficos

De los 62 asmáticos incluidos, 74 % eran hombres y 25 % eran mujeres; y la edad promedio fue de 11,3±2,8 años, con un rango entre 6 y 16. El 96,8 % de los participantes vivía en zonas urbanas y el 3,2 % en zonas rurales. En cuanto al IMC, se observó que el 78,3 % de los pacientes estaba en los rangos de la normalidad, el 6,7 % tenía bajo peso y, el 15,0 %, sobrepeso; no se encontraron pacientes con obesidad. Solo el 16,7 % de los pacientes informó convivir con fumadores.

### Aspectos clínicos de los pacientes pediátricos tratados con inmunoterapia

El 96,8 % de los pacientes sufría rinitis asociada con el asma, el 27,4 %, de dermatitis atópica, y el 61,3 %, de conjuntivitis alérgica. El promedio de edad al inicio de los síntomas respiratorios era de 1,7 años, y había antecedente de bronquiolitis en 27,4 % de ellos, definida esta como la presencia de sibilancias antes de los tres años; en el análisis de este subgrupo de pacientes, no se encontraron diferencias estadísticamente significativas.

Al inicio de la inmunoterapia, la sensibilización predominante fue con *D. pteronyssinus* y *D. farinae,* en ambos casos con el 93,6 %, seguida por sensibilización con *B. tropicalis* en el 58, 1 %. El 62,9 % de los pacientes estaba en inmunoterapia con dos alérgenos y el 33,9 % con tres, en tanto que el 3,2 % había comenzado con dos y había cambiado a tres alérgenos después de las primeras seis dosis. La vía subcutánea se empleaba en el 90,3 % de los casos, y dos de los pacientes evaluados habían iniciado con administración sublingual y pasaron a la subcutánea en el curso de la aplicación de las seis primeras dosis.

Se evaluó la percepción de la mejoría con la inmunoterapia en un rango de 0 a 100 %, usando la escala visual análoga: 0 % correspondía a ausencia de mejoría y 100% a una completa mejoría de los síntomas bronquiales. Se encontró que el 9,75 % (n=6) de los pacientes tuvo una mejoría por debajo del 50 %, el 48,4 % (n=30), una entre el 50 y el 80 %, y la mejoría en el 41,9 % (n=26) de los pacientes fue igual o mayor del 90 %. El promedio de dosis de inmunoterapia recibidas hasta el momento de la evaluación fue de 28 ± 12,5, con un rango entre 6 y 54.

### Efecto de la inmunoterapia según el ACT, la GINA y la espirometría

Al evaluar el efecto clínico de la inmunoterapia en el puntaje del ACT, se encontró que, antes de iniciarla, 30 pacientes tenían asma no controlada, 28 tenían un buen control y en 4 pacientes la enfermedad estaba totalmente controlada. El 30,0 % (n=9) de los 30 pacientes con asma no controlada continuó en esta condición después de la inmunoterapia, el 46,7 % (n=14) logró un buen control y el 23,3 % (n=7) alcanzó un control total de la enfermedad.

El 10,7 % (n=3) de los 28 pacientes con buen control del asma empeoraron después de la inmunoterapia según el puntaje del ACT, que los calificaba con asma no controlada, el 42,9 % (n=12) continuó con un buen control, en tanto que el 46,4 % (n=13) logró un control total del asma.

Cuando se evaluó a los cuatro pacientes que tenían un control total del asma al inicio, se encontró que ninguno de ellos presentó asma no controlada, en tanto que el puntaje del ACT del 50 % (n=2) disminuyó y llegaron a un buen control, y el 50 % (n=2) continuó con un control total.

**Figura 2:**
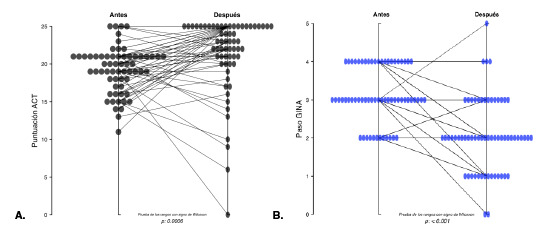
Efecto de la inmunoterapia según: **A.** el puntaje del ACT y **B.** el de la GINA

El 19,4 % (n=12) de los 62 pacientes evaluados después del tratamiento con inmunoterapia, tuvo puntajes del ACT correspondientes a asma no controlada, el 45,2 % (n=28) logró un buen control del asma y el 35,5 % (n=22) alcanzó un puntaje de 25, es decir, un control total del asma (cuadro 1). Estos resultados fueron estadísticamente significativos (p=0,00006) (figura 2A).

**Cuadro 1:** Efecto de la inmunoterapia según la puntuación del ACT

Puntuación del ACT (McNemar-Bowker; p=0,096	Antes de la inmunoterapia		Después del tratamiento con inmunoterapia					
			Asma no controlada n (%)		Buen control del asma n (%)		Control total del asma n (%)	
	18 dosis n (%)	Más de 18 dosis n (%)	6-18 dosis	Más de 18 dosis	6-18 dosis	Más de 18 dosis	6-18 dosis	Más de 18 dosis
Asma no controlada	9 (100)	21 (100)	2 (22,2)	7 (23,3)	4 (44,4)	10 (47,6)	3 (33,3)	4 (19)
Buen control del asma	11 (100)	17 (100)	2 (18,2)	1 (5,9)	4 (36,4)	8 (47,1)	5 (45,5)	8 (47,1)
Control total del asma	1 (100)	3 (100)	0 (0,0)	0 (0,0)	1 (100)	1 (33,3)	0 (0)	2 (66,7)
Total	21 (100)	41 (100)	4 (19)	8 (19,5)	9 (42,9)	19 (46,3)	8 (38,1)	14 (34,1)

### Efecto de la inmunoterapia en el puntaje del ACT según subgrupos

Se hizo un análisis por subgrupos con base en el número de dosis de inmunoterapia recibidas hasta el momento de la evaluación, siguiendo criterios establecidos en la literatura especializada que sugieren que la respuesta clínica a mejoría clínica con la inmunoterapia se aprecia principalmente entre las 12 y las 18 dosis ^15^. Los pacientes se agruparon en aquellos con menos de 18 dosis y aquellos con más de 18 dosis.

### Efecto de la inmunoterapia en la condición clínica según el ACT en pacientes con 6 a 18 dosis

Un total de 21 pacientes había recibido menos de 18 dosis hasta el momento de la evaluación. De ellos, nueve tenían diagnóstico de asma no controlada según el ACT y, de estos, el 22,2 % (n=2) mantenía esta condición en el momento de evaluar la inmunoterapia, el 44,4 % (n=4) logró un buen control del asma y el 33,3 % (n=3) tuvo puntajes indicativos de un control total del asma.

Se encontró que 11 de los 21 pacientes tenían un buen control del asma antes de iniciar la inmunoterapia y, de ellos, el 18,2 % (n=2) presentaba asma no controlada en el momento de evaluar la inmunoterapia, 36,4 % (n=4) continuaba teniendo un buen control del asma y un 45,5 % (n=5) logró el control total.

Un solo paciente con menos de 18 dosis de inmunoterapia tenía control total de asma antes de iniciarla y, en el momento de la evaluación, tuvo puntajes del ACT que indicaban un buen control del asma.

El 19 % (n=4) de los 21 pacientes con menos de 18 dosis no logró el control del asma según los puntajes del ACT en el momento de la evaluación de la inmunoterapia, el 42,9 % (n=9) tuvo un buen control y el 38,1 % (n=8), un control total del asma. Estos resultados no fueron estadísticamente significativos (p=0,096) (cuadro 1).

### Efecto de la inmunoterapia según la puntuación del ACT en pacientes con más de 18 dosis

De los 62 pacientes del estudio, 41 habían recibido más de 18 dosis hasta el momento de la evaluación, 21 de ellos con asma no controlada. De estos 21, el 23,3 % (n=7) continuaba en la misma condición en el momento de la evaluación, el 47,6 % (n=10) alcanzó un buen control del asma y el 19 % (n=4) logró su control total.

Del grupo de 41, en 17 pacientes con un buen control del asma según el puntaje del ACT previo a la inmunoterapia, en el momento de la evaluación, se encontró que: en 5,9 % (n=1), disminuyeron los puntajes del ACT y el asma se consideró como no controlada; en 47,1 % (n=8), continuó el buen control del asma, y en 47,1 % (n=8), se logró el control total del asma.

De los 41 pacientes evaluados en este subgrupo, el 19,5 % (n=8) tenía asma no controlada en el momento de la evaluación, el 46,3 % (n=19) un buen control de la enfermedad y el 34,1 % (n=14) alcanzó el control total del asma. Los resultados fueron estadísticamente significativos (p= 0,001) (cuadro 1).

### Efecto clínico de la inmunoterapia según el puntaje de resultados terapéuticos de la GINA

Al evaluar el tratamiento para el asma según el grado de la GINA, se encontró que ningún paciente se encontraba en el paso 1 de tratamiento, 19,4 % (n=12) estaba en el paso 2, el 46,8 % (n=29) en el paso 3, el 33,8 % (n=21) en el paso 4 y ningún paciente estaba en el paso 5. En la evaluación posterior al inicio de la inmunoterapia, el 3,22 % (n=2) no recibía ningún medicamento para el asma, el 22,6 % (n=14) estaba en el paso 1, el 45,2 % (n=28) en el paso 2, el 22,6 % (n=14) en el paso 3, el 4,8 % (n=3) en el paso 4 y el 1,6 % (n=1) en el paso 5 (cuadro 2). Se observó que antes del inicio de la inmunoterapia la mayoría de los pacientes evaluados (80,6 %) estaba en los pasos 3 y 4 de gradación de la GINA, y en el momento de la evaluación posterior, la mayoría (67,6 %) estaba en los pasos 1, 2 y 3. Estos resultados fueron estadísticamente significativos (p=0,001) (figura 2B).

**Cuadro 2:** Efecto de la inmunoterapia según el escalón terapéutico de la estrategia de la GINA

Escalón terapéutico estrategia de la GINA (p<0,001)	Antes de la inmunoterapia n (%)	Después del tratamiento con inmunoterapia					
		Paso 0 n (%)	Paso 1 n (%)	Paso 2 n (%)	Paso 3 n (%)	Paso 4 n (%)	Paso 5 n (%)
Paso 2	12 (100)	0 (0,0)	5 (41,7)	3 (25,0)	4 (33,3) 0	(0,0)	0 (0,0)
Paso 3	29 (100)	2 (6,9)	6 (20,7)	13 (44,8)	7 (24,1) 0	(0,0)	1 (3,4)
Paso 4	21 (100)	0 (0,0)	3 (14,3)	12 (57,1)	3 (14,3) 3	(14,3)	0 (0,0)
Total	62 (100)	2 (3,2)	14 (22,6)	28 (45,2)	14 (22,6) 3	(4,8)	1 (1,6)

### Efecto de la inmunoterapia según la espirometría antes y después del uso de broncodilatador

Según el volumen espiratorio forzado en un segundo (VEF1) y el porcentual, la mediana de los valores anteriores al uso de los agonistas beta-2 antes de la inmunoterapia era de 1,52 L y, después, de 1,87 L, con un valor de p<0,0001 y un tamaño de efecto de -0,34 (-0,48 a -0,17). Los valores del VEF1 posteriores al uso de los agonistas beta-2 fueron de 1,66 L antes del inicio de la inmunoterapia y, después, de 1,87 L, con un valor de p=0,0005 y un tamaño de efecto de -0,28 (-0,43 a -0,11). Al evaluar los valores de VEF1 porcentual antes y después de la inmunoterapia, no se hallaron diferencias estadísticamente significativas y los tamaños del efecto fueron casi nulos. El cambio en el VEF1 tanto porcentual como numérico (litros) después de la inmunoterapia no fue estadísticamente significativo al compararlo con los valores previos (cuadro 3).

**Cuadro 3:** Efecto de la inmunoterapia según la espirometría previa y posterior al uso de broncodilatador

	Antes Me (RI)	Después Me (RI)	p	Tamaño del efecto rbis (I.C. 95%)
VEF_1_ previo (L)	1,52 (0,70)	1,87 (1,07)	<0,0001	-0,34 (-0,48 a -0,17)
VEF_1_ posterior (L)	1,66 (0.64)	1,87 (1.13)	0,0005	-0,28 (-0,43 a -0,11)
VEF_1_ previo %	96 (15)	96 (19)	0,8393	0,01 (-0,17 a 0,19)
VEF_1_ posterior %	99 (16)	98,5 (17)	0,8022	0,07 (-0,11 a 0,24)
% cambio en el VEF_1_	3,5 (9)	4,5 (10)	0,7862	0,04 (-0,14 a 0,22)

Me: mediana; RI: rango Intercuartilico; rbis: correlación biserial

### Evaluación del efecto de la inmunoterapia en la población de estudio

Esta evaluación tuvo en cuenta dos variables: el puntaje del ACT y el grado de la GINA. El 79 % (n=49) de los pacientes mostró mejoría después de la inmunoterapia según la puntuación del ACT y el grado de la GINA, es decir, esta tuvo un impacto positivo. En la evaluación por subgrupos, los menores de 10 años no presentaron cambios significativos en los puntajes comparados con los mayores de 10 años. En cuanto a las comorbilidades, el 80 % de los pacientes con rinitis registraron mejoría en el puntaje del ACT y el grado de la GINA, como también sucedió en el 76,5 % de aquellos con dermatitis atópica y en el 79,8 % de aquellos con conjuntivitis. El 72,2 % de los pacientes sensibilizados con *B. tropicalis* y un 81 % de los sensibilizados con *D.pteronyssinus* presentaron mejores puntajes en el ACT y la GINA, y aunque el porcentaje de cambio en ambos casos fue menor en los pacientes sensibilizados con *B. tropicalis* frente a los sensibilizados con *D. pteronyssinus,* no se encontraron diferencias estadísticamente significativas (cuadro 4). 

**Cuadro 4:** Evaluación del efecto de la inmunoterapia en población pediátrica con diagnóstico de asma

Variables		Impacto de la inmunoterapia			
		Efecto positivo (%)	Efecto negativo (%)	p	RP (IC_95%_)
Sexo	Hombres	38 (82,6)	8 (17,4)	0,255	1,20 (0,84 a 1,71)
	Mujeres	11 (68,8)	5 (31,3)		
Grupo de edad (años)	≤10	20 (76,9)	6 (23,1)	0,730	0,95 (0,73 a 1,24)
	≥10	29 (80,6)	7 (19,4)		
Estrato económico	Bajo	16 (76,2)	5 (23,8)	0,696	0,95 (0,71 a 1,26)
	Medio-alto	33 (80,5)	8 (19,5)		
Rinitis	Sí	48 (80,0)	12 (20,0)	0,355	1,60 (0,40 a 6,43)
	No	1 (50,0)	1 (50,0)		
Dermatitis	Sí	13 (76,5)	4 (23,5)	0,763	0,96 (0,71 a 1,29)
	No	36 (80,0)	9 (20,0)		
Conjuntivitis	Sí	30 (78,9)	8 (21,1)	0,984	0,99 (0,77 a 1,30)
		19 (79,2)	5 (20,8)		
Sensibilización con *B. tropicalis*	Sí	26 (72,2)	10 (27,8)	0,111	0,82 (0,64 a 1,04)
	No	23 (88,5)	3 (11,5)		
Sensibilización con *D. farinae*	Sí	46 (79,3)	12 (20,7)	0,841	1,06 (0,59 a 1,89)
	No	3 (75,0)	1 (25,0)		
Sensibilización con *D. pteronyssinus*	Sí	47 (81,0)	11 (19,0)	0,181	1,62 (0,60 a 4,35)
	No	2 (50,0)	2 (50,0)		
Tipo de inmunoterapia	Con dos alergenos	32 (82,1)	7 (17,9)	0,592	1,07 (0,81 a 1,43)
	Con tres alergenos	16 (76,2)	5 (23,8)		

## Discusión

Las personas con asma alérgica a los ácaros del polvo doméstico, uno de los alérgenos más comunes involucrados en esta enfermedad ^16^, presentan exacerbaciones recurrentes, aumento de la gravedad y disminución de la función pulmonar. La inmunoterapia con alérgenos específicos es la única que ha demostrado eficacia en la modulación persistente del curso natural de esta enfermedad, por lo cual se recomienda en varias guías para tratar el asma alérgica ^4^.

En el presente estudio se seleccionaron 62 participantes que cumplían con los criterios de selección entre los 150 pacientes del estudio RATTA. La selección se hizo por conveniencia ^12^^)^ porque se apuntaba más a una inferencia científica que a una de representatividad poblacional estadística; por ello, los participantes debían cumplir con criterios muy específicos para garantizar la calidad de la información obtenida. Los resultados se basaron en tres elementos de verificación principales: el puntaje del ACT, el grado de la GINA y los cambios del VEF1 en la espirometría, además de la evaluación de la percepción de los pacientes con respecto a la mejoría mediante una escala visual análoga. La combinación del puntaje del ACT y el grado de la GINA buscó determinar el efecto de la inmunoterapia y los análisis de subgrupos se orientaron a delimitar el número de dosis de inmunoterapia hasta el momento de la evaluación presencial, ya que este varía en un rango amplio entre 6 y 54 dosis, como ya se mencionó en la metodología.

La división en dos grupos se basó en lo establecido en la literatura, es decir, el número de dosis con el que se espera una mejoría clínica es de 12 a 18. De los 62 pacientes evaluados, 30 tenían asma no controlada antes del inicio de la inmunoterapia, 7 (23,3 %) habían logrado un control total de la enfermedad en el momento de la evaluación, y 10 (47,6), un buen control. De los 28 pacientes con buen control del asma, 12 (42,9 %) continuaron teniéndolo y 13 lograron un control total de la enfermedad. Es decir, 50 pacientes de los 62 evaluados tenían un buen control o control total del asma en el momento de la evaluación.

Los resultados fueron estadísticamente significativos y hubo una mejoría de el puntaje del ACT. Al evaluar los subgrupos según el número de dosis de inmunoterapia (hasta 18 dosis y más de 18 dosis), se encontraron diferencias importantes también con los puntajes del ACT, con una tendencia al buen control o control total del asma en el momento de la evaluación. Estos datos no alcanzaron una significación estadística en el subgrupo de hasta 18 dosis. Sin embargo, debe aclararse que el tamaño de la muestra en este subgrupo (n=22) era pequeño, lo que pudo ocasionar que no se lograran valores de p significativos, ello sin menospreciar la mejoría importante de los datos obtenidos en el subgrupo con más de 18 dosis de inmunoterapia.

Al evaluar según la gradación de la GINA, se encontró que, antes de la inmunoterapia, el 80 % de los pacientes se encontraba en los grados 3 o 4 de la GINA y que, en la evaluación posterior, el 67,7 % se encontraba en los grados 3 o 4 y solo uno (4,8 %) estaba en el grado 4. Estos datos fueron estadísticamente significativos (p=0,001). Se presentaron cambios importantes en el porcentaje de pacientes con un resultado terapéutico de grado 3 (46,8 a 22,6 %) o de grado 4 (33,8 a 4,8 %) (figura 2), y que avanzaron a los grados 1 y 2. Estos cambios en la gradación del tratamiento según la GINA antes y después de la inmunoterapia, se reflejaron en la disminución del número de medicamentos y las dosis utilizadas para controlar el asma, lo que se correlaciona con la mejoría en el puntaje del ACT en cuanto a la sintomatología.

Estos resultados son similares a los de otros estudios, por ejemplo, el metanálisis de Abramson, *et al.,* en el cual se analizaron 42 estudios clínicos aleatorizados sobre la inmunoterapia subcutánea contra los ácaros del polvo casero. Se encontró que este tratamiento redujo los síntomas del asma y disminuyó la hiperreactividad bronquial ^17^. En la revisión sistemática de Dhami, *et al.,* que incluyó 51 estudios sobre las inmunoterapias subcutánea y sublingual en niños y adultos, se encontró que estos tratamientos pueden reducir en forma significativa el puntaje en las escalas de síntomas a corto plazo y sin producir reacciones adversas graves, en el asma alérgica inducida por ácaros del polvo doméstico ^18^.

Cuando se evaluó el efecto de la inmunoterapia combinando la puntuación del ACT y la gradación de la GINA, el 79 % (n=49) de los pacientes evidenció mejoría, lo que se considera como un impacto positivo. Este resultado no se vio influenciado por condiciones como la edad, las comorbilidades asociadas o las condiciones sociodemográficas. En general, hubo una correlación de este efecto positivo determinado de manera objetiva con los datos sobre la percepción de la efectividad de la inmunoterapia clasificada por los pacientes mediante la escala visual análoga, pues el 45,2 % (n=28) respondió que entre 50 y 80 %, y el 41,9 % (n=26) respondió que igual o mayor de 90 %. Por lo tanto, hubo una correlación con el porcentaje de pacientes que mostraron mejoría en los puntajes, lo que permite pensar que los resultados obtenidos mostraron un efecto en general positivo objetivado mediante el puntaje del ACT y la gradación de la GINA. No se encontraron cambios estadísticamente significativos en los valores de VEF1 previos y posteriores al uso de broncodilatadores y antes de iniciar la inmunoterapia, comparados con los registrados en el momento de la evaluación. Asimismo, se observó que los valores porcentuales del VEF1, previos y posteriores, se mantuvieron estables en los dos momentos de la inmunoterapia.

El porcentaje de sensibilización a los ácaros del polvo casero fue de más del 90 % con *D. farinae* y *D. pteronyssinus,* lo que coincide con lo reportado en la literatura mundial ^19^. Además, el 60 % de los pacientes presentó sensibilización a *B. tropicalis,* lo que es alto comparado con lo reportado en estudios como el de Sánchez, *et al.,* en el cual el 39 % de los participantes mostraron sensibilización a este alérgeno ^20^. Solo 33 % de los pacientes pediátricos en el presente estudio estaba recibiendo inmunoterapia con extracto de tres alérgenos, el otro 62 % recibía extracto con dos alérgenos. Se cree que está prevalencia en la sensibilización comparada con la de otros países europeos o norteamericanos es más alta en Colombia por ser un país situado en el trópico ^21^.

En comparación con otros estudios, el presente es uno de los que más pacientes pediátricos ha evaluado ^18^. Por otra parte, en el estudio de Yepes, *et al.,* se evaluó el efecto de la inmunoterapia con *D. farinae* y *D. pteronyssinus* sobre la calidad de vida de pacientes con rinitis y asma alérgica en una muestra colombiana mediante dos encuestas validadas. Al año de seguimiento, se observó que un porcentaje considerable presentaba cambios positivos en términos de calidad de vida, aunque no se evaluaron los cambios en los puntajes de resultados terapéuticos. Este estudio, sin embargo, tuvo la limitación de contar con una reducida muestra de pacientes ^10^.

En cuanto a nuestro estudio, las limitaciones incluyeron el número de pacientes, aunque ya se mencionó este apuntaba más a una inferencia científica que a una inferencia estadística de representatividad poblacional. Además, tampoco hubo un grupo de control para determinar el efecto de la inmunoterapia en el asma, y fue amplio el rango de las dosis empleadas hasta el momento de la evaluación. Por último, en futuros trabajos de este mismo tipo, sería importante usar otras escalas diferentes a la del ACT y la GINA, pues la inmunoterapia subcutánea no solo impacta en la sintomatología, sino en la calidad de vida, la disminución del uso de medicamentos, etc.

La inmunoterapia específica con alérgenos sigue siendo un tratamiento relevante que permite disminuir el uso de medicamentos y el número de exacerbaciones en pacientes con asma alérgica. En la población pediátrica ha demostrado tener un efecto positivo y estadísticamente significativo, con una mejoría cercana al 60 %. No se logró evidenciar un efecto importante en la mejoría de los valores del VEF1 en la espirometría asociados con la inmunoterapia. Sin embargo, debe recordarse que, para que un paciente pueda iniciarla, debe tener una espirometría con un VEF1 mínimo de más del 70 %. Además, se ha establecido, la relevancia de los ácaros del polvo casero como fuente de exacerbación de la enfermedad. En el presente estudio, se pudo evidenciar un efecto positivo, según la escala del ACT y la de resultados terapéuticos de la GINA.
